# Altered functional connectivity strength in chronic insomnia associated with gut microbiota composition and sleep efficiency

**DOI:** 10.3389/fpsyt.2022.1050403

**Published:** 2022-11-22

**Authors:** Ziwei Chen, Ying Feng, Shumei Li, Kelei Hua, Shishun Fu, Feng Chen, Huiyu Chen, Liping Pan, Caojun Wu, Guihua Jiang

**Affiliations:** ^1^Jinan University, Guangzhou, China; ^2^Department of Medical Imaging, Guangdong Second Provincial General Hospital, Guangzhou, China; ^3^Department of Radiology, Affiliated Hospital of Chengdu University, Chengdu, China; ^4^The Second School of Clinical Medicine, Southern Medical University, Guangzhou, China

**Keywords:** chronic insomnia, gut microbiota, resting-state fMRI, functional connectivity strength, brain function

## Abstract

**Background:**

There is limited evidence on the link between gut microbiota (GM) and resting-state brain activity in patients with chronic insomnia (CI). This study aimed to explore the alterations in brain functional connectivity strength (FCS) in CI and the potential associations among altered FCS, GM composition, and neuropsychological performance indicators.

**Materials and methods:**

Thirty CI patients and 34 age- and gender-matched healthy controls (HCs) were recruited. Each participant underwent resting-state functional magnetic resonance imaging (rs-fMRI) for the evaluation of brain FCS and was administered sleep-, mood-, and cognitive-related questionnaires for the evaluation of neuropsychological performance. Stool samples of CI patients were collected and subjected to 16S rDNA amplicon sequencing to assess the relative abundance (RA) of GM. Redundancy analysis or canonical correspondence analysis (RDA or CCA, respectively) was used to investigate the relationships between GM composition and neuropsychological performance indicators. Spearman correlation was further performed to analyze the associations among alterations in FCS, GM composition, and neuropsychological performance indicators.

**Results:**

The CI group showed a reduction in FCS in the left superior parietal gyrus (SPG) compared to the HC group. The correlation analysis showed that the FCS in the left SPG was correlated with sleep efficiency and some specific bacterial genera. The results of CCA and RDA showed that 38.21% (RDA) and 24.62% (CCA) of the GM composition variation could be interpreted by neuropsychological performance indicators. Furthermore, we found complex relationships between Alloprevotella, specific members of the family Lachnospiraceae, Faecalicoccus, and the FCS alteration, and neuropsychological performance indicators.

**Conclusion:**

The brain FCS alteration of patients with CI was related to their GM composition and neuropsychological performance indicators, and there was also an association to some extent between the latter two, suggesting a specific interaction pattern among the three aspects: brain FCS alteration, GM composition, and neuropsychological performance indicators.

## Introduction

Chronic insomnia (CI), a frequent condition in adults, is the most common sleep disorder in the human population. CI exhibits diverse manifestations, including difficulties in initiating or maintaining sleep and obtaining refreshing sleep, as well as a hyperarousal state (1, 2). Persistent insomnia disorder has been associated with chronic conditions like hypertension (3) but also with cancer (4, 5). In addition, CI can impair social, cognitive, and behavioral functioning, resulting in a high risk of suicide (6, 7) and fatal traffic accidents (8). Therefore, CI significantly impacts human physical and mental health and impairs social development, and thus has become a major public health issue worldwide (9). However, the neural mechanisms of CI have not been fully elucidated.

The potential link between sleep disorders and gut microbiota (GM) has drawn considerable interest in recent years (10–13). Studies have found that the microbial diversity and relative abundance (RA) of specific GM differed significantly between insomnia patients and healthy individuals (11, 12). Li et al. further showed that Faecalibacterium and Blautia can be used as iconic bacteria to distinguish patients with CI and healthy controls (HCs), whereas the combination of Lachnospira and Bacteroides was most helpful for identifying patients with acute insomnia (13). A study found that sleep quality and the severity of insomnia were the main factors that drove the variation in microbiome community structure in patients with major depressive disorder (14). Another study found RA changes in some members of the microbiota following two nights of partial sleep deprivation (15). Using redundancy analysis (RDA), Liu et al. found that the GM profile at the phylum level was highly correlated with clinical sleep parameters, including polysomnography (PSG) parameters, total sleep time, sleep efficiency, and sleep latency (10), which indicated that GM composition alterations could be explained by clinical sleep parameters. The findings of the above studies suggest that the GM is closely related to insomnia disorder. Some specific flora may be potential prognostic markers of insomnia symptoms or even subtypes. The microbiota-gut-brain axis (MGBA) is a well-known bidirectional communication system that has shown great potential in exploring the neural mechanisms of CI. The GM dysbiosis regulates brain physiology, cognition, and behavior *via* various pathways, including metabolic, endocrine, and immune signaling pathways (16–19). Bacterial metabolites, such as short-chain fatty acids (SCFAs), bioactive compounds, or neuroactive metabolites, can affect neuronal architecture and function (20). However, research on whether the GM has an impact on insomnia through the MGBA is still in its infancy, and further studies are needed.

Resting-state functional magnetic resonance imaging (rs-fMRI) is an effective means of studying the MGBA in humans and is currently an essential technique for studying the underlying neural mechanisms of insomnia (21). Rs-fMRI can be used to observe the link between the GM and brain function and perhaps even explain how the GM affects resting-state brain activity (22). Rs-fMRI has been applied in several studies investigating the potential brain-gut interaction mechanisms in healthy individuals (23, 24) and those with neuropsychiatric disorders (25–27), including schizophrenia and amnestic mild cognitive impairment. Rs-fMRI offers a unique perspective for studying MGBA communication pathways in insomnia. Functional connectivity strength (FCS), a graph theory-based data-driven method calculated by degree centrality (DC), can quantify the strength of network nodes with high connectivity to neighboring brain regions and thus identify communication hubs in the human brain (28–30). The FCS method based on rs-fMRI can be used to directly compute global brain connectivity and visualize important architectural topology abnormalities in the brain functional connectome at the voxel level, thus reflecting the functional interactions of the brain in the resting state (31). Previous studies based on this method showed that the brain areas with increased FCS or DC in insomnia patients were mainly located in the right superior parietal lobe, insula, cerebellum posterior lobe, precuneus, and left middle frontal gyrus. In contrast, reduced FCS or DC values were mainly located in the bilateral frontal lobe, temporal lobe, right inferior parietal gyrus, insula, right occipital lobe, and right cerebellum anterior lobe (29, 32–35). With great potential in the study of GM-brain function interaction pathways in insomnia patients, the FCS method, which is accurate and highly reproducible, could compensate for the shortcomings of traditional functional connectivity analyses (33, 36). However, to our knowledge, no investigation has combined an evaluation of brain FCS and the intestinal flora to explore brain-gut communication mechanisms in CI patients.

This study aimed to investigate the interconnections among the three aspects of brain function, the GM, and neuropsychological performance indicators in patients with CI. We hypothesized that altered brain function in patients with CI is related to GM composition and that both are related to neuropsychological changes. To test this hypothesis, in addition to recruiting CI patients and HCs for evaluating neuropsychological performance and studying intergroup brain functional alterations based on the FCS method of rs-fMRI, we collected stool samples from the CI group to analyze the GM composition in CI patients and its potential association with brain FCS alteration and neuropsychological performance indicators. An in-depth understanding of the relationship among the altered brain function, microbiota, and neuropsychological performance of patients with CI may be used to screen for new GM-neuroimaging markers for the study of neural mechanisms of CI and to explore potential future intervention targets.

## Materials and methods

### Participants

**FIGURE 1:**
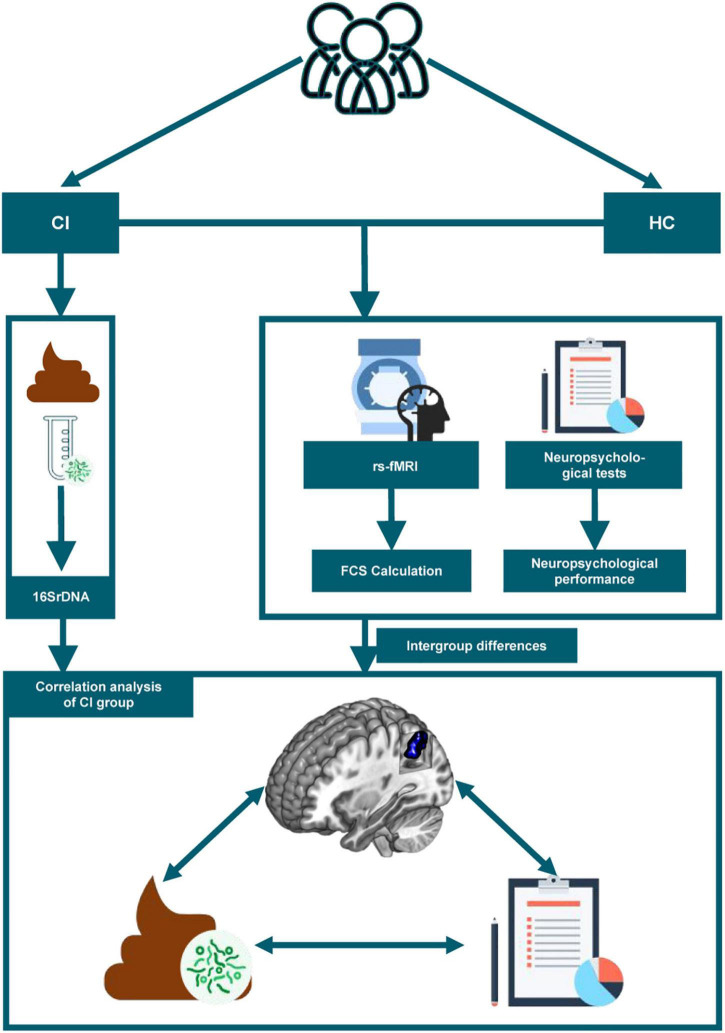
The study flow chart is depicted. CI, chronic insomnia; HC, healthy controls; rs-fMRI, resting-state functional magnetic resonance imaging; FCS, functional connectivity strength.

Informed consent was obtained from each participant according to the principles of the Declaration of Helsinki. Ethical approval was obtained from the Ethics Committee of Guangdong Second Provincial General Hospital. All our tests were completed on the day of the patient’s first visit. The stools were collected on the morning of the visit and the neuropsychological assessments were performed prior to the MRI scans. The flow chart of our whole study was shown in Figure 1.

The inclusion criteria for patients with CI were as follows: diagnosis of CI based on the criteria in the Diagnostic and Statistical Manual of Mental Disorders, version 5 (DSM-V); free of other sleep disorders; and no history of receiving cognitive behavioral therapy or medication. The diagnosis of CI was confirmed by two psychiatrists with >15 years of clinical experience. A third opinion was sought to reach a consensus diagnosis when disagreement happened. HCs were recruited through local advertisements with the following inclusion criteria: (a) good sleep quality without difficulty falling or maintaining asleep; and (b) no history of psychiatric or neurological disorders. In addition, participants were excluded if the following criteria were met: (a) T1-weighted and T2-weighted fluid-attenuated inversion-recovery (T2-FLAIR) weighted sequences identifying organic brain disease or a history of head trauma; (b) a history of transient ischemic attack or stroke; (c) the presence of other systematic diseases, including severe respiratory, cardiovascular, renal, liver, or endocrine diseases; (d) pregnancy, lactation, or menstruation in females; (e) a history of smoking or drug/alcohol abuse; (f) the use of any psychotropic drug or medication that could affect sleep; (g) a diagnosis of irritable bowel syndrome; (h) diarrhea while participating in the study or the use of antibiotics, probiotics, and prebiotics before sample collection; and (i) rotating shift work or crossing more than one-time zone within 2 weeks before the study. According to the strict inclusion/exclusion criteria, we excluded three patients due to the lack of access to fresh fecal samples collection. Two patients were excluded after MRI scanning due to the presence of abnormal hyperintense signals on T2-FLAIR images. One healthy individual was excluded because of falling asleep during scanning. Finally, 30 patients with CI and 34 HCs were included. The two groups were matched by age, sex, and education. All participants were right-handed and aged 18–60 years.

Before the MRI scans, neuropsychological tests were administered by trained psychiatrists to all participants to evaluate their sleep quality, emotional state, and general cognitive level. These tests included: the Pittsburgh Sleep Quality Index (PSQI) and Insomnia Severity Index (ISI) for the evaluation of sleep; the Self-Rating Depression Scale (SDS), Self-Rating Anxiety Scale (SAS), and Hamilton Anxiety Scale (HAMA) for the evaluation of emotional state; and the Montreal Cognitive Assessment (MoCA), Digit Symbol Test, and Digit Symbol Substitution Test (DSST) for the evaluation of the general cognitive level. In addition, information on sleep efficiency (the percentage of time asleep while in bed at night) and total sleep time was collected using answers to individual questions within the PSQI. The above evaluation of neuropsychological performance was carried out in total by two trained psychiatrists in a face-to-face, one-to-one approach.

### Collection of fecal samples and gut microbiota analysis

Fresh fecal samples were collected from CI patients into sterile fecal boxes and stored immediately in −80°C freezers to avoid air oxidation and urine contamination during transport. The subsequent 16SrDNA high-throughput sequencing was performed on the MiSeq platform. First, DNA was extracted from fecal samples using a QIAamp DNA Mini Kit (QIAGEN, Hilden, Germany). After quality control, the amplification of the 16S rDNA variable region, library construction, sequencing, and subsequent data analyses were performed. Then QIIME2 software^1^ was used to filter out the low-quality reads and obtain the remaining high-quality clean data. FLASH (37) (Fast Length Adjustment of Short, FLASH, v1.2.11) software was used to assemble paired-end reads for obtaining high-quality sequences. After that, chimeras were removed using the UCHIME (38) algorithm based on VSEARCH.

Operational taxonomic units (OTUs) were clustered by UPARSE software at 97% similarity in the next step. The OTU representative sequence was compared with the template sequence from the Ribosomal Database Project (RDP) (39) for species annotation using a confidence threshold of 0.8. After OTU classifications, stacked bar figures were generated from the phylum- and genus -level RA, to visually display the GM composition of CI patients.

### fMRI data acquisition, preprocessing, and functional connectivity strength calculation

#### fMRI data acquisition

All participants were scanned on a Philips 3.0 T MRI scanner. Image acquisition was performed by two trained radiographers. Participants laid supine with their head fixed by foam pads and body fixed by straps. Noise-canceling earplugs were provided to minimize scanning noise. During scanning, participants were asked to remain quiet and to avoid thinking about anything particular. The radiographers communicated with the participants through a microphone to be sensitive to any discomfort that the subjects might have during the examination. Communication only takes place when necessary. Finally, self-reports were collected after scanning to assess patient cooperation. In the presence of a poor degree of patient cooperation, we rescanned the participant again to obtain new rs-fMRI datasets for further analysis.

Imaging parameters for high-resolution anatomical imaging were as follows: TR/TE: 7.6/3.6 ms; 256 × 256 matrix; FOV: 256 × 256 mm^2^; flip angle: 8°; and 185 axial slices with no gap. Rs-fMRI data were obtained *via* gradient-recalled echo-planar imaging (EPI) with the following parameters: TR/TE: 2,000/30 ms; 64 × 61 matrix; FOV: 224 × 224 mm^2^; flip angle: 90°; and 33 slices obtained using an interleaved slice acquisition sequence with a 1.0 mm gap. The scanning time for each participant was approximately 8 min with 240 time points. T1-WI and T2-FLAIR images were used to detect organic brain disease or a history of head trauma.

#### Data preprocessing and functional connectivity strength calculation

Data preprocessing was performed in MATLAB by DPABI_V4.3 software.^2^ First, initial functional images were excluded to allow magnetic field stabilization and the participants’ acclimation to the scanner environment. Then, time layer correction and motion correction were conducted. Data with head movement displacement > 1.5 mm or rotation angle > 1.5° were excluded. The remaining functional images were spatially normalized into a standard EPI template from the Montreal Neurological Institute (MNI) and resampled to 3 mm isotropic voxels. Next, the resampled images were smoothed by a 4 mm full-width half-maximum Gaussian kernel and detrended to remove the linear signal drift. After this, nuisance covariates were removed by regression. All image data were filtered with a bandpass filter on a frequency range of 0.01–0.08 to remove the effects of noise.

For preprocessed rs-fMRI data, FCS values were computed by the DPARSF.^3^ First, for each participant, the Pearson correlation of time series between each pair of voxels was calculated to construct the brain functional connectivity matrix. Then, Pearson correlation coefficients were corrected by Fisher’s r-to-z transformation to make the data meet the normal distribution.

For each voxel, FCS was computed as the summed weights of its connections with the remaining voxels of the brain (40). The correlation threshold for the FCS calculation was set at 0.25 (41, 42) to eliminate the influence of noise.

### Statistical analysis

Statistical analyses for demographic data of the participants were processed with R software (R Studio software, version 4.1.2) and SPSS 25.0 (SPSS Inc., Chicago, IL, United States). The Shapiro–Wilk test was applied to test whether the data satisfied a normal distribution. Continuous variables are presented as the mean value ± standard deviation (if normally distributed), and comparisons of the two groups were carried out with independent sample *t-*test. The Mann–Whitney *U* test was used to compare groups when the data for continuous variables were not normally distributed. The comparison of sex between groups was performed with a χ^2^ test.

To explore the voxel-based FCS differences between CI patients and HCs, two independent sample *t-*tests with age, gender, education, and head movement as covariates were performed in DPABI software. Two-tailed Gaussian random field theory (GRF) was used to correct for multiple comparisons (voxel level *p* < 0.005, cluster level *p* < 0.05). At last, the mean FCS value of brain regions with significant differences was extracted for subsequent analysis. Then, we used Spearman rank correlation for the correlation analysis between FCS alterations and neuropsychological performances in CI patients. The clinical indicators of neuropsychological performance included in the correlation analysis were those with significant group differences and sleep parameters related to the altered brain FCS in the CI group.

To further demonstrate whether neuropsychological performance indicators related to CI could explain the GM composition, in addition to creating stacked bar charts of the microbiota composition, we performed RDA or CCA for the RAs in patients with CI. The clinical indicators for neuropsychological performance included in the RDA and CCA analysis were as described above. Constrained ordination technique and detrended correspondence analysis (DCA) were used to determine the chosen method. The results of DCA and the specific selection principles of RDA or CCA are provided in the Supplementary Table 1. Then, the correlation between the RAs of GM and, neuropsychological performance indicators, and the RAs of GM and abnormal FCS were analyzed by Spearman’s rank correlation, respectively. *P-*values were corrected for multiple inferences using the Benjamini-Hochberg false discovery rate (FDR) procedure (43). Finally, the heatmaps (the absolute value of *r* > 0.3, significance level *p* < 0.05) were plotted by R software to visualize the results.

## Results

### Demographic and clinical characteristics

The analysis of the general data of participants showed no significant differences (all *p* > 0.05) in age, gender, or years of education between groups. Sleep assessments (PSQI and ISI), mood assessments (SAS, SDS, and HAMA), and total MoCA scores representing cognition were significantly different between the CI and HC groups (*p* < 0.05). Table 1 shows the general characteristics of the participants.

**TABLE 1:** Demographics and clinical characteristics of CI patients and HCs.

Characteristic	CIs (*N* = 30)	HCs (*N* = 34)	Statistics	*P*-value
Age (y)	39.50 (26.50∼49.75)	35.00 (29.00∼48.50)	527.00^c^	0.82
Gender (F/M)	7/23	5/29	1.00^b^	0.57
Education (y)	15.50 (12.00∼16.00)	16.00 (15.25∼16.00)	412.50^c^	0.13
PSQI (score)	12.40 ± 2.98	4.35 ± 1.89	12.71^a^	<0.01*
ISI (score)	16.50 ± 5.35	4.14 ± 1.28	12.34^a^	<0.01*
SAS (score)	50.47 ± 9.31	40.32 ± 8.31	4.57.00^a^	<0.01*
SDS (score)	54.00 (48.00∼61.00)	41.00 (38.00∼43.00)	877.00^c^	<0.01*
HAMA (score)	18.50 ± 7.91	2.53 ± 1.05	10.98	<0.01*
MoCA (score)	25.00 (23.00∼27.00)	28.00 (26.25∼29.00)	274.50^c^	0.01*
DSST (*n*)	45.50 (37.00∼63.75)	57.00 (48.00∼64)	392.50^c^	0.12
DST (*n*)	13.83 ± 2.61	13.65 ± 1.82	0.33^a^	0.75
Sleep efficiency (%)	72.57 ± 15.75	/	/	/
Total sleep time (h)	6.00 (5.00∼6.00)	/	/	/

^a^Independent two sample *t*-test; ^b^χ^2^ test; ^c^Mann–Whitney *U* test; *p < 0.05.

CIs, chronic insomnia patients; HCs, healthy controls; PSQI, Pittsburgh Sleep Quality Index; ISI, Insomnia Severity Index; SAS, Self-Rating Anxiety Scale; SDS, Self-Rating Depression Scale; HAMA, Hamilton Anxiety Scale; MoCA, Montreal Cognitive Assessment; DSST, Digit Symbol Substitution Test; DST, Digit Span Test; y, year; h, hour; min, minute; s, second.

### Relationships between brain functional connectivity strength alteration and neuropsychological performance indicators

**FIGURE 2:**
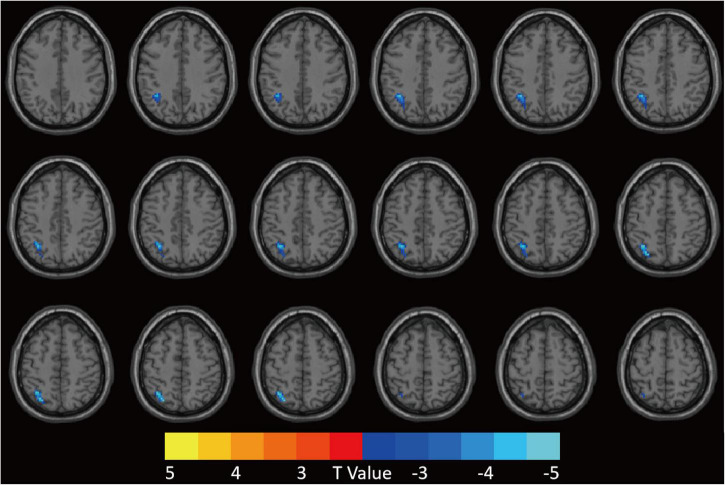
Patients with CI showed decreased FCS value in the left SPG compared to HCs. GRF was used for multiple comparisons, setting the height threshold at *p* < 0.005 and the clustering threshold at *p* < 0.05. CI, chronic insomnia; FCS, functional connectivity strength; SPG, superior parietal gyrus; HCs, healthy controls; GRF, Gaussian random fields.

**FIGURE 3:**
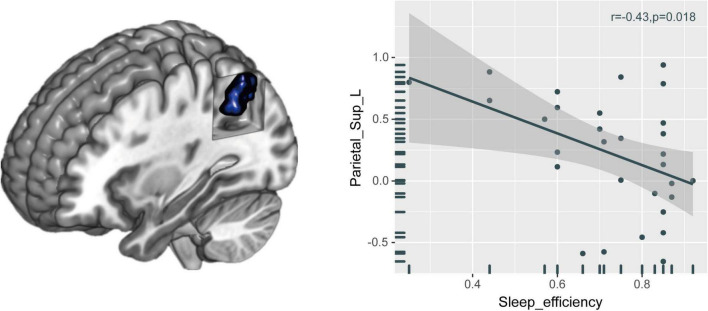
Relationships between brain FCS alteration and neuropsychological performances in patients with CI. The FCS value of the left SPG was negatively correlated with sleep efficiency. FCS, functional connectivity strength; CI, chronic insomnia; SPG, superior parietal gyrus.

The FCS value of the left superior parietal gyrus (SPG) was significantly reduced in the CI group compared with the HC group (Figure 2 and Table 2). The correlation analysis showed that the FCS value of the left SPG was negatively correlated with sleep efficiency (*r* = −0.430, *p* = 0.018), while no significant correlations were found between the FCS value of the left SPG and other clinical indicators for neuropsychological performance (Figure 3).

**TABLE 2:** Brain regions with abnormal FCS in CI group compared with HCs.

Brain area	Cluster size	Peak MNI coordinate			Peak *t*-value	*P*-value
		x	y	z		
Parietal_Sup_L	109	−30	−66	51	−4.1618	<0.05

FCS, functional connectivity strength; CI, chronic insomnia; HCs, healthy controls; Sup, superior; L, left; MNI, Montreal Neurological Institute.

### Gut microbiota composition of chronic insomnia patients

**FIGURE 4:**
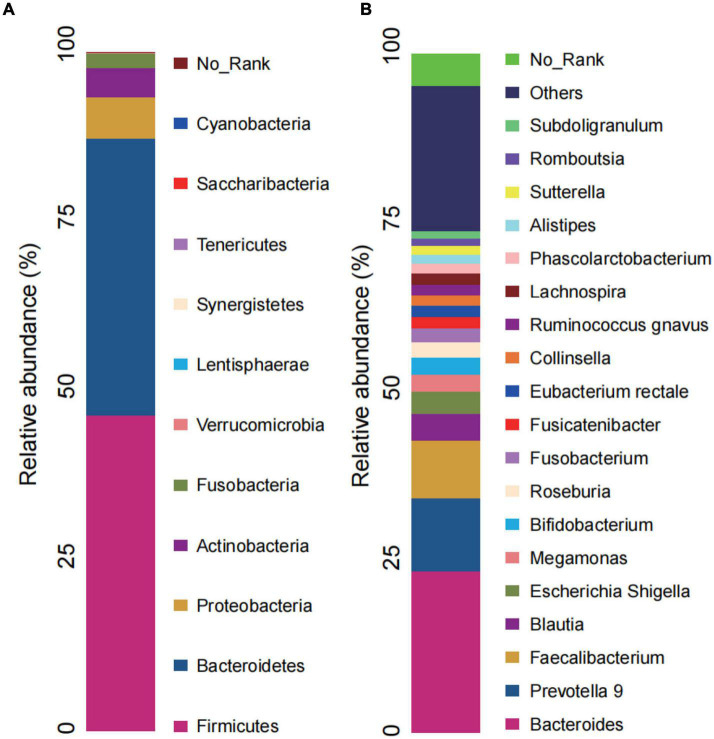
Microbial composition in CI. **(A)** Relative abundance bar charts of GM in the CI group at the phylum level. **(B)** Relative abundance bar charts of GM in the CI group at the genus level. GM, gut microbiota; CI, chronic insomnia.

The RAs of intestinal bacteria at the phylum and genus levels are shown in Figure 4. Firmicutes and Bacteroidetes were the dominant bacterial phyla in the CI group, accounting for 46.50 and 40.78%, respectively. At the genus level, the predominant genera in CI patients were Bacteroides, Prevotella 9, Faecalibacterium, and Blautia. RAs at other levels in CI patients are shown in the Supplementary Figure 1.

### Gut microbiota composition was highly correlated with neuropsychological performance indicators in the chronic insomnia group

**FIGURE 5:**
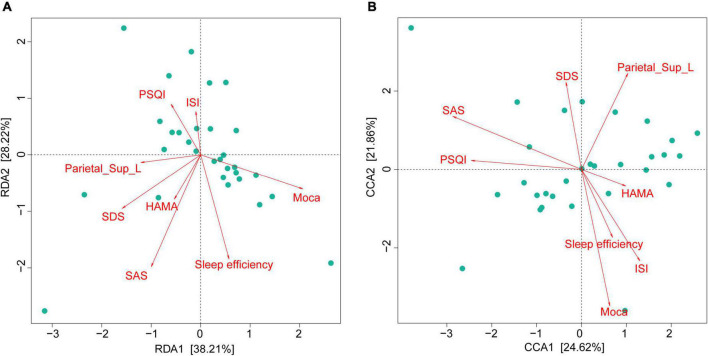
Gut microbiota composition strongly associated with neuropsychological performances in CI patients. RDA (at the phylum level) and CCA (at the genus level) demonstrated that 38.21% **(A)** and 24.62% **(B)** of the variance could be interpreted by seven environmental factors (in other words: neuropsychological performances). CI, chronic insomnia; RDA, redundancy analysis; CCA, canonical correspondence analysis.

We found 38.21% (RDA) and 24.62% (CCA) of the variance could be interpreted by seven environmental factors, in other words, neuropsychological performance indicators, including sleep efficiency, PSQI, ISI, SAS, SDS, HAMA, and MoCA scores (Figure 5).

### Associations between gut microbiota, regional functional connectivity strength alteration, and neuropsychological performance indicators

**FIGURE 6:**
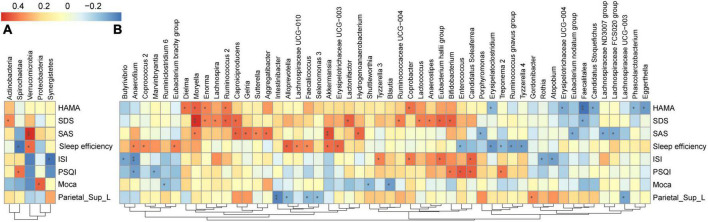
Heatmap of the associations between GM and altered brain FCS and neuropsychological performances in CI patients. Heat maps at the phylum level **(A)** and genus level **(B)** demonstrate the correlation coefficients between these variables. Blue, negative correlation; red, positive correlation. *p < 0.05, **p < 0.01. GM, gut microbiota; FCS, functional connectivity strength; CI, chronic insomnia.

As shown in Figure 5, we found that the GM composition at phylum level and genus level were both partially microbially significantly correlated with the neuropsychological performance indicators. The RA of GM in CI patients at the phylum level showed no significant correlation with reduced FCS in the left SPG (Figure 6A); however, significant correlations were found at the genus level (Figure 6B). At the genus level, Alloprevotella (*r* = −0.366, *p* = 0.047), Intestinibacter (*r* = −0.486, *p* = 0.007), Lachnospiraceae_UCG-003 (*r* = −0.416, *p* = 0.022), Gordonibacter (*r* = 0.362, *p* = 0.049), Faecalicoccus (*r* = −0.371, *p* = 0.043), and Selenomonas_3 (*r* = −0.363, *p* = 0.049) were significantly correlated with the reduced FCS value of the left SPG. After FDR adjustment (*q-*value < 0.05), Intestinibacter (*q-*value = 0.048), Lachnospiraceae_UCG-003 (*q-*value = 0.048), and Faecalicoccus (*q-*value = 0.049) were still significantly correlated. At the same time, Alloprevotella, Gordonibacter, and Selenomonas_3 showed a trend for a significant correlation (*q-*value = 0.050) with the reduced FCS value of the left SPG. In the dominant florae, we found a negative correlation between Blautia and MoCa score (see Supplementary Table 2). In addition, we also found significant correlations between other specific non-dominant genera and some indicators of neuropsychological performance as well as altered FCS value.

Among the aforementioned taxa, three taxa, namely, Alloprevotella, specific members of family Lachnospiraceae, and Faecalicoccus, were also significantly associated with neuropsychological performance indicators: Alloprevotella (*r* = 0.445, *p* = 0.014), Faecalicoccus (*r* = 0.361, *p* = 0.050), and Lachnospiraceae_UCG-010 (*r* = 0.3795, *p* = 0.039) were positively correlated with sleep efficiency, and Lachnospiraceae_FCS020_group (*r* = −0.4078, *p* = 0.025) and Lachnospiraceae_ ND3007_group (*r* = −0.4038, *p* = 0.027) were negatively correlated with the SAS score. The correlation coefficients and FDR-corrected *p*-values for the significant results of the correlation analysis between each bacterium at the phylum and genus levels and neuropsychological performance indicators are shown in Supplementary Table 2.

## Discussion

In the present study, we used the FSC method based on rs-fMRI to measure abnormal brain functional connectivity at the voxel level. The CI group showed lower FCS in the left SPG than the HC group. The decreased FCS in the left SPG was significantly correlated with sleep efficiency and specific intestinal microbiota. Specifically, the composition of Intestinibacter, Lachnospiraceae_UCG-003, and Faecalicoccus were significantly correlated with the FCS alteration in the left SPG. It is worth noting that after FDR adjustment, there were still complex relationships between Alloprevotella and specific members of the family Lachnospiraceae, Faecalicoccus and the FCS alteration and neuropsychological performance indicators. These findings together suggested a complex association among three aspects: altered regional FCS, neuropsychological performance indicators, and specific intestinal microbiota. This research is the first, as far as we are aware, to evaluate the relationship between the SPG and the GM profile. Our findings may provide new neuroimaging evidence for MGBA interaction mechanisms in CI.

### Functional connectivity strength alteration in the left superior parietal gyrus in chronic insomnia patients

In our study, the FCS value of the left SPG was significantly reduced in the CI group. In patients with primary insomnia, the presence of weakened connectivity between the right SPG and the superior frontal gyrus and other brain regions (44) and between the bilateral superior parietal lobule and superior frontal gyrus (45) was confirmed, supporting our finding of reduced FCS value in the SPG. Previous studies have shown that altered SPG function is complexly correlated with sleep quality and cognitive changes, including mood, memory, and attention (46–50). After sleep restriction, the functional connectivity variability of the SPG and thalamus was reduced and was significantly correlated with insomnia severity (51). This finding is consistent with the significant negative correlation between the reduced FCS values in the left SPG and sleep efficiency in our study. In a recent study, Li et al. (32) found increased DC values in the right post-central gyrus, rolandic operculum, insula, and SPG in CI patients using real-time fMRI neurofeedback. An interventional study by Huang et al. (52) also found that applying 1 Hz low-frequency repetitive transcranial magnetic stimulation of the parietal cortex was practical for the improvement of anxiety and insomnia symptoms in patients with generalized anxiety disorder and insomnia. The findings of the studies mentioned above longitudinally corroborated the relationship between the parietal cortex and insomnia, suggesting that the potential mechanism of the involvement of the SPG in insomnia may be due to the impaired ability of CI patients to regulate top-down attention and several memory processes. While significant differences in DSST or DST assessments between insomnia patients and HCs were not always found in previous studies (53, 54), it is not surprising that our study did not show differences in memory and attention assessments between groups. The differences in sample sizes could contribute to the discordant results. However, taken together, the FCS changes in the CI group and their correlation with sleep efficiency in our study were supported, suggesting that the SPG is somehow involved in sleep regulation and that its functional alteration may be an important neurobiological change in CI patients.

### Associations among the gut microbiota, altered functional connectivity strength, and neuropsychological performance indicators

In the present study, Firmicutes and Bacteroidetes were the dominant phyla in the CI group, as supported in other literature (12). The predominant genera in CI patients were Bacteroides, Prevotella 9, Faecalibacterium, and Blautia. The genera above have also been shown to be significantly different between HCs and CI patients (11–13). Therefore, although GM data from the HC group were not collected to assess differences between baseline in our study, the CI group may have dominant bacteria that differ from those of the HC group.

The results of the subsequent CCA and RDA implied that the GM profile was highly correlated with the neuropsychological performance indicators of CI patients, suggesting that neuropsychological performance indicators could explain the GM composition (10). Our study showed results that were similar to those of a previous study (10), and the results of the genus-level CCA provided additional support for the correlation of flora with neuropsychological performance indicators. Follow-up Spearman correlation analysis results showed complicated relationships between the reduced value FCS value of the left SPG and genus-level bacteria. Although the exact mechanism is unknown, to the best of our knowledge, significant correlations between Intestinibacter, Lachnospiraceae_UCG-003, and Faecalicoccus bacteria and left SPG function in CI patients have never been reported before. Interestingly, among the bacteria associated with FCS changes in the SPG, Alloprevotella, specific members of the Lachnospiraceae family, and Faecalicoccus were also correlated with mood assessment or sleep assessment scores after strict FDR correction. This finding indicates that mood and sleep alterations in CI patients may be related to supraparietal gyrus function, and GM composition also might be involved in sleep regulation in some way.

Intestinibacter was found to be correlated with the reduced FCS of the left SPG in CI patients. The latest study found that Intestinibacter was significantly correlated with sleep phenotypes, including chronotype and sleep duration, in different brain aging patterns (55), and another study also found that Intestinibacter was directly related to sleep quality independent of depression severity (14). However, no previous studies have explored the relationship between Intestinibacter and changes in brain function in patients with CI, while our study found an association between Intestinibacter and left SPG function in CI patients. In summary, the present study found that the RA of Intestinibacter was strongly associated with the FCS in the left SPG and that this association was independent of neuropsychological performance, suggesting that Intestinibacter might somehow be involved in the neural mechanisms of altered brain function in insomnia.

Alloprevotella and Lachnospiraceae were also found to be associated with functional alterations of the left SPG in CI patients. Alloprevotella and Lachnospiraceae are closely associated with the production of SCFAs (e.g., significant positive correlation with metabolic changes in butyrate) (56, 57) and amino acid metabolism (56). SCFAs can penetrate the blood-brain barrier, increase neurogenesis, and improve neuronal homeostasis and function (58). SCFAs are thought to have a wide range of effects on neural and behavioral processes (58) and may mediate the relationship between gut bacteria and the neural mechanisms of sleep (59, 60). For example, butyrate, according to the neurochemical basis of the hypersomnia-hyperarousal theory (61), is a source of sleep- and wakefulness-related signals. Increasing butyrate by using GABA-rich fermented milk or other means can increase non-rapid eye movement sleep and affect sleep duration and sleep onset latency in mice (62). Therefore, we hypothesized that Alloprevotella and Lachnospiraceae bacteria could affect brain function in CI patients by modulating SCFA production. In addition, our study found that some members of the Lachnospiraceae family were significantly associated with both mood assessment and sleep assessment scores in the CI group. Several previous studies have also implied the interaction of specific Lachnospiraceae family members with sleep deprivation, sleep fragmentation, and insomnia severity (11, 63–65). The abovementioned findings suggest that Alloprevotella and Lachnospiraceae may be involved in the MGBA through SCFA metabolism, affecting insomnia performance and mood changes.

The results of the correlation analysis also showed a correlation between FCS changes in the SPG and the genera Faecalicoccus/Faecalitalea, which are all members of the family Erysipelotrichaceae. Several studies have shown that Erysipelotrichaceae is associated with not only sleep (15, 66) but also organismal metabolism, such as lipid metabolism, in humans and rodents (67–69). Sleep loss (including sleep restriction, sleep deprivation, and sleep disruption) triggers GM-related abnormalities in organismal metabolism (64, 70), for example, sleep deprivation may be an important step in oxidative stress and adenosine triphosphate depletion (71), which Erysipelotrichaceae is associated with (72). There have also been studies showing that Erysipelotrichaceae can influence neurological inflammation. Autoimmune encephalomyelitis enhances the response of T helper 17 cells in the gut (73, 74). In addition, the RA of Erysipelotrichaceae has been associated with impaired cognitive function due to other diseases (75) like spatial memory performance before and after treatment in patients with phenylketonuria (73). The aforementioned studies provided support for those of our study, in which the genus Faecalicoccus of the family Erysipelotrichaceae was positively correlated with sleep efficiency and the genus Faecalitalea was negatively correlated with SDS. On the basis of these results combined with the results of our study, we speculate that Erysipelotrichaceae family members communicate with the parietal gyrus through the endocrine metabolic and inflammatory stress pathways of the MGBA, which may be the underlying neurobiological basis for altered cognitive functions such as mood changes in CI patients.

Thus, the complex relationships that exist among GM, including Alloprevotella, Lachnospiraceae, and Faecalicoccus; FCS changes in the left SPG; and neuropsychological performance indicators in CI patients may represent a way in which the GM communicates with local brain regions through SCFA metabolism and inflammatory stress pathways and further affects patients’ neuropsychological performance.

### Limitations

Nevertheless, there are several limitations of our work. First, this was a cross-sectional study. Therefore, a longitudinal study is needed to determine whether therapeutic interventions affect stool microbiota structure in CI patients or, conversely, whether specific interventions such as microbiota transplantation or probiotic therapies can improve the brain function and symptoms of insomnia patients. Second, we were unable to control for all possible confounders that may affect GM composition. The participants included in this study were all local residents with relatively consistent dietary habits who were advised by the researchers to eat lightly and avoid a stimulating diet before the experiment. The inclusion and exclusion criteria were closely followed to minimize the effects on the composition of the GM. In addition, 16SrDNA amplicon sequencing was used in this study. Although the results of metagenomic sequencing may be more comprehensive, 16SrDNA amplicon sequencing can ensure the credibility of the results at a lower cost and is widely used in similar types of research. Moreover, we did not evaluate the baseline difference in GM composition between the groups, as performed in other studies (10, 13). However, to avoid important omissions, we performed RDA and CCA to analyze the correlation between environmental factors (neuropsychological performance indicators) and the microbial community (GM composition), as reported in the previous literature. We also performed a correlation analysis of FCS value in the abnormal brain region and the GM profile in CI patients to explore in detail the potential relationship among brain function changes, GM composition, and neuropsychological performance in CI. Finally, the FCS method calculated by degree centrality was used to explore the abnormal brain areas in CI patients without combining this method with brain structural methods based on 3D-T1WI, DTI, etc. However, our team has explored brain structural alterations and the mechanism of CI in the past (53, 76, 77). In the future, we will further explore the complex mechanism of the interaction between brain functional and structural alterations and the GM in CI patients to screen for more accurate imaging biomarkers for MGBA interaction mechanisms.

## Conclusion

In conclusion, we found a complex association between specific GM composition, FCS abnormalities (left SPG), and neuropsychological performance indicators in patients with CI. Notably, to the best of our knowledge, this research showed a link between left SPG functional alterations and specific GM profile in patients with CI, which has not previously been reported. Alloprevotella, several members of the Lachnospiraceae family, and Faecalicoccus were significantly correlated or trended toward correlating with altered SPG function and neuropsychological performance indicators. These findings have substantial implications for screening new GM-neuroimaging markers for the study of neural mechanisms of CI and exploring potential future intervention targets for its treatment.

## Data availability statement

The original contributions presented in the study are publicly available. This data can be found here: https://www.ncbi.nlm.nih.gov/bioproject/PRJNA882369. Further inquiries can be directed to jgh2023@outlook.com.

## Ethics statement

The studies involving human participants were reviewed and approved by Ethics Committee of the Guangdong Second Provincial General Hospital. The patients/participants provided their written informed consent to participate in this study. Written informed consent was obtained from the individual(s) for the publication of any potentially identifiable images or data included in this article.

## Author contributions

GJ and YF designed the study. ZC and YF coordinated subject enrollment and data collection. ZC performed the analysis, interpreted the data, and wrote the first draft of the manuscript. ZC, KH, SF, and FC assisted in the several procedures of fMRI data preprocessing and FCS calculation. YF, SL, and GJ contributed to the final revision of the manuscript. GJ took full responsibility for this study, including the study design and the decision to publish the manuscript. All authors contributed to the article and approved the submitted version.

## Footnotes

1
https://qiime2.org/

2
http://rfmri.org/dpabi

3
http://rfmri.org/dparsf
