# Supplementary material for: Altered functional connectivity strength in chronic insomnia associated with gut microbiota composition and sleep efficiency
Source: Front Psychiatry. 2022 Nov 22;13:1050403. doi: 10.3389/fpsyt.2022.1050403 (PMC9722753; doi:10.3389/fpsyt.2022.1050403)
Supplement: Supplementary file 1 [file Data_Sheet_1.zip › Editorial 1.DOCX]

**Supplementary materials**

**Table S1. Results of detrended correspondence analysis.**

**Phylum level**

|  | DCA1 | DCA2 | DCA3 | DCA4 |
| --- | --- | --- | --- | --- |
| Eigenvalues | 0.1425 | 0.04790 | 0.07509 | 0.08235 |
| Decorana values | 0.2214 | 0.03321 | 0.02063 | 0.01369 |
| Axis lengths | **1.3165** | 0.66019 | 0.86648 | 0.95685 |

**Genus level**

|  | DCA1 | DCA2 | DCA3 | DCA4 |
| --- | --- | --- | --- | --- |
| Eigenvalues | 0.4739 | 0.1382 | 0.2037 | 0.11829 |
| Decorana values | 0.4994 | 0.2002 | 0.1323 | 0.09004 |
| Axis lengths | **4.0499** | 1.7299 | 1.7749 | 1.47713 |

*Abbreviations*: DCA, Detrended Correspondence Analysis.

The principle of RDA or CCA selection: firstly, DCA analysis is performed on the species composition data, then observe the value of the first column of Axis lengths in the analysis result. If it is greater than 4.0, CCA is selected; if it is between the range 3.0-4.0, both RDA and CCA are selected, and CCA is selected by default; if it is less than 3.0, RDA analysis should be selected. In this study, the value of the first column of Axis lengths at phylum level bacteria was <3, while the value of the first column of Axis lengths at genus level bacteria was >4. Therefore, RDA was selected for phylum-level bacteria, and CCA was selected for genus-level bacteria.

**Fig. S1. Bar graphs of relative abundance at class, order and family levels of gut microbiota in chronic insomnia patients.**


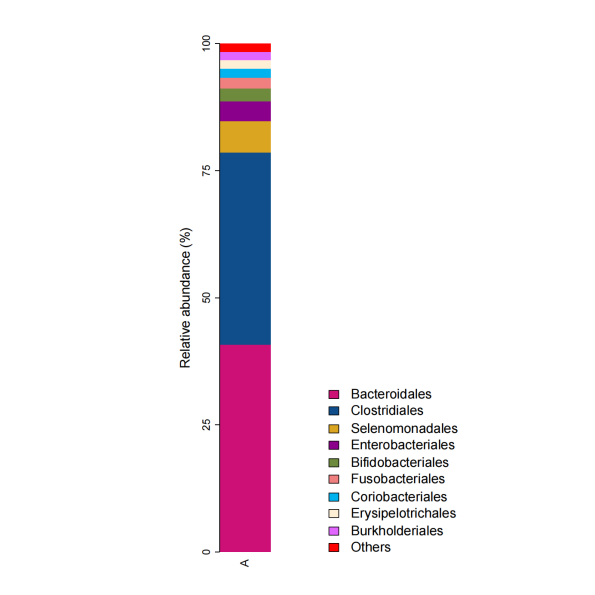

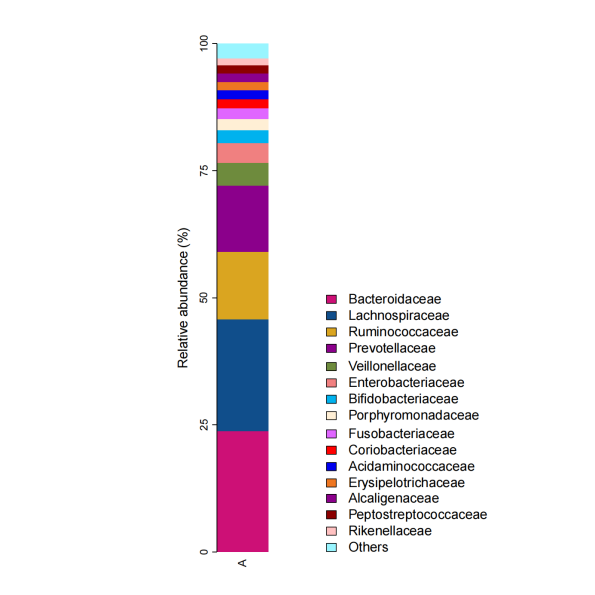

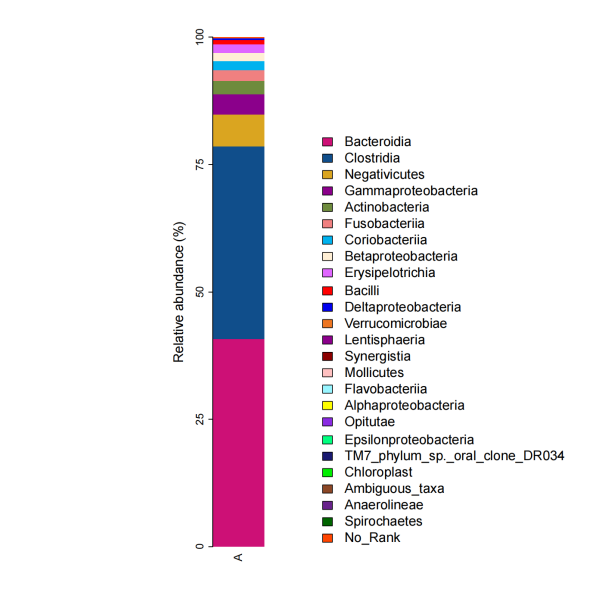


**Table S2. Phylum level and genus level Spearman’s correlation coefficients and FDR corrected P values (q values).**

| **Table S2a**  Spearman’s correlation coefficients and FDR corrected P values (q values) at Phylum level. | | | | |
| --- | --- | --- | --- | --- |
| Taxon | Factor | Cor | P value | q value |
| Verrucomicrobia | SAS | 0.507421959 | 0.004207948 | 0.028 |
| Proteobacteria | MoCa | 0.424455031 | 0.019396824 | 0.048 |
| Verrucomicrobia | sleep_efficiency | 0.405072266 | 0.026380844 | 0.048 |
| Spirochaetae | PSQI | 0.395944912 | 0.030316781 | 0.048 |
| Synergistetes | ISI | -0.383255764 | 0.036567406 | 0.048 |
| Spirochaetae | sleep_efficiency | -0.368515445 | 0.045088686 | 0.048 |
| Actinobacteria | SDS | 0.363859623 | 0.048086433 | 0.048 |

| **Table S2b**  Spearman’s correlation coefficients and FDR corrected P values (q values) at Genus level. | | | | |
| --- | --- | --- | --- | --- |
| Taxon | Factor | Cor | P value | q value |
| Faecalitalea | HAMA | -0.538184921 | 0.002156787 | 0.0478 |
| Moryella | HAMA | 0.439583178 | 0.0150747 | 0.0478 |
| Eggerthella | HAMA | -0.43132977 | 0.017320624 | 0.0478 |
| Dielma | HAMA | 0.410455861 | 0.024262011 | 0.0478 |
| Coprobacter | HAMA | 0.409537534 | 0.024613235 | 0.0478 |
| Candidatus_Stoquefichus | HAMA | -0.401060211 | 0.028056167 | 0.0478 |
| Erysipelotrichaceae_UCG-004 | HAMA | -0.401060211 | 0.028056167 | 0.0478 |
| Ruminococcus_2 | HAMA | 0.394950333 | 0.030773029 | 0.0482 |
| Enorma | HAMA | 0.379678762 | 0.038505282 | 0.0488 |
| Erysipelatoclostridium | HAMA | -0.374929133 | 0.041204982 | 0.0489 |
| Phascolarctobacterium | HAMA | -0.37003334 | 0.044144212 | 0.0489 |
| Anaerofilum | ISI | -0.466365447 | 0.009384291 | 0.0478 |
| Candidatus_Soleaferrea | ISI | 0.44301672 | 0.014214602 | 0.0478 |
| Atopobium | ISI | -0.425682308 | 0.019011823 | 0.0478 |
| Eubacterium_hallii_group | ISI | 0.418654747 | 0.021304512 | 0.0478 |
| Butyrivibrio | ISI | -0.409606195 | 0.024586832 | 0.0478 |
| Rothia | ISI | -0.405540139 | 0.026190908 | 0.0478 |
| Coprobacter | ISI | 0.396016451 | 0.030284177 | 0.0482 |
| Tyzzerella_3 | ISI | 0.37079434 | 0.043676674 | 0.0489 |
| Blautia | MoCa | -0.437876534 | 0.015518062 | 0.0478 |
| Shuttleworthia | MoCa | -0.374095718 | 0.04169395 | 0.0489 |
| Enterococcus | PSQI | 0.458104418 | 0.010904238 | 0.0478 |
| Candidatus_Soleaferrea | PSQI | 0.434500659 | 0.01642703 | 0.0478 |
| Anaerofilum | PSQI | -0.428379615 | 0.018187818 | 0.0478 |
| Bifidobacterium | PSQI | 0.402445321 | 0.02746826 | 0.0478 |
| Treponema_2 | PSQI | 0.395944912 | 0.030316781 | 0.0482 |
| Marvinbryantia | PSQI | -0.393943717 | 0.031240454 | 0.0482 |
| Intestinibacter | PARIETAL_SUP_L | -0.48566376 | 0.006514476 | 0.0478 |
| Lachnospiraceae_UCG-003 | PARIETAL_SUP_L | -0.41557414 | 0.022378866 | 0.0478 |
| Faecalicoccus | PARIETAL_SUP_L | -0.371279179 | 0.043380868 | 0.0489 |
| Akkermansia | SAS | 0.477175568 | 0.007668345 | 0.0478 |
| Caproiciproducens | SAS | 0.438979896 | 0.015230204 | 0.0478 |
| Hydrogenoanaerobacterium | SAS | 0.428210708 | 0.018238536 | 0.0478 |
| Gelria | SAS | 0.420120341 | 0.020808495 | 0.0478 |
| Aggregatibacter | SAS | 0.41978291 | 0.020921845 | 0.0478 |
| Lachnospiraceae_FCS020_group | SAS | -0.407768975 | 0.025301365 | 0.0478 |
| Lachnospiraceae_ND3007_group | SAS | -0.403859393 | 0.026878458 | 0.0478 |
| Eubacterium_nodatum_group | SAS | -0.401993503 | 0.027658919 | 0.0478 |
| Porphyromonas | SAS | -0.392827157 | 0.031765648 | 0.0487 |
| Moryella | SAS | 0.38412018 | 0.036111084 | 0.0488 |
| Sutterella | SAS | 0.380006656 | 0.038324279 | 0.0488 |
| Moryella | SDS | 0.542094821 | 0.001972152 | 0.0478 |
| Ruminococcus_2 | SDS | 0.444447539 | 0.013868463 | 0.0478 |
| Lactonifactor | SDS | 0.442383953 | 0.014369959 | 0.0478 |
| Eubacterium_hallii_group | SDS | 0.435367769 | 0.016189444 | 0.0478 |
| Bifidobacterium | SDS | 0.434875108 | 0.016324079 | 0.0478 |
| Caproiciproducens | SDS | 0.426328275 | 0.018811731 | 0.0478 |
| Faecalitalea | SDS | -0.400114835 | 0.028463281 | 0.0478 |
| Lactococcus | SDS | 0.389998148 | 0.0331284 | 0.0488 |
| Ruminococcaceae_UCG-004 | SDS | 0.387467596 | 0.034387082 | 0.0488 |
| Anaerostipes | SDS | 0.383261182 | 0.036564531 | 0.0488 |
| Enorma | SDS | 0.370670657 | 0.043752391 | 0.0489 |
| Lachnospira | SDS | 0.369535732 | 0.044452082 | 0.0489 |
| Alloprevotella | sleep_efficiency | 0.444712328 | 0.013805183 | 0.0478 |
| Erysipelatoclostridium | sleep_efficiency | -0.444503156 | 0.013855151 | 0.0478 |
| Ruminococcus_gnavus_group | sleep_efficiency | -0.439799395 | 0.015019289 | 0.0478 |
| Tyzzerella_4 | sleep_efficiency | -0.431925358 | 0.017149776 | 0.0478 |
| Erysipelotrichaceae_UCG-003 | sleep_efficiency | 0.428703138 | 0.018091 | 0.0478 |
| Akkermansia | sleep_efficiency | 0.41997077 | 0.020858676 | 0.0478 |
| Eubacterium_brachy_group | sleep_efficiency | 0.385758961 | 0.035258573 | 0.0488 |
| Lachnospiraceae_UCG-010 | sleep_efficiency | 0.379562842 | 0.038569435 | 0.0488 |
| Anaerofilum | sleep_efficiency | 0.378098589 | 0.039387211 | 0.0488 |
| Enterococcus | sleep_efficiency | -0.377995058 | 0.039445554 | 0.0488 |
| Treponema_2 | sleep_efficiency | -0.368515445 | 0.045088686 | 0.0492 |
| Alloprevotella | PARIETAL_SUP_L | -0.366061692 | 0.046649454 | 0.0500 |
| Ruminiclostridium_6 | MoCa | -0.363697233 | 0.048193777 | 0.0500 |
| Gordonibacter | PARIETAL_SUP_L | 0.36262622 | 0.048906521 | 0.0500 |
| Selenomonas_3 | PARIETAL_SUP_L | -0.362530113 | 0.048970886 | 0.0500 |
| Faecalicoccus | sleep_efficiency | 0.361431963 | 0.049711114 | 0.0500 |

*Abbreviations*: FDR, False Discovery Rate; PSQI, Pittsburgh Sleep Quality Index; ISI, Insomnia Severity Index; SAS, Self-Rating Anxiety Scale; SDS, Self-Rating Depression Scale; HAMA, Hamilton Anxiety Scale; MoCA, Montreal Cognitive Assessment; Sup, superior; L, left.
